# Dirofilariasis mouse models for heartworm preclinical research

**DOI:** 10.3389/fmicb.2023.1208301

**Published:** 2023-06-22

**Authors:** A. E. Marriott, J. L. Dagley, S. Hegde, A. Steven, C. Fricks, U. DiCosty, A. Mansour, E. J. Campbell, C. M. Wilson, F. Gusovsky, S. A. Ward, W. D. Hong, P. O'Neill, A. Moorhead, S. McCall, J. W. McCall, M. J. Taylor, J. D. Turner

**Affiliations:** ^1^Department of Tropical Disease Biology, Centre for Drugs and Diagnostics, Liverpool School of Tropical Medicine, Pembroke Place, Liverpool, United Kingdom; ^2^TRS Laboratories Inc, Athens, GA, United States; ^3^Department of Infectious Diseases, College of Veterinary Medicine, University of Georgia, Athens, GA, United States; ^4^Eisai Global Health, Cambridge, MA, United States; ^5^Department of Chemistry, University of Liverpool, Liverpool, United Kingdom

**Keywords:** dirofilariasis, heartworm, *Wolbachia*, pharmacology, parasitology, symbiosis, one health, drug development

## Abstract

**Introduction:**

Dirofilariasis, including heartworm disease, is a major emergent veterinary parasitic infection and a human zoonosis. Currently, experimental infections of cats and dogs are used in veterinary heartworm preclinical drug research.

**Methods:**

As a refined alternative *in vivo* heartworm preventative drug screen, we assessed lymphopenic mouse strains with ablation of the interleukin-2/7 common gamma chain (γc) as susceptible to the larval development phase of *Dirofilaria immitis*.

**Results:**

Non-obese diabetic (NOD) severe combined immunodeficiency (SCID)γc^−/−^ (NSG and NXG) and recombination-activating gene (RAG)2^−/−^γc^−/−^ mouse strains yielded viable *D. immitis* larvae at 2–4 weeks post-infection, including the use of different batches of *D. immitis* infectious larvae, different *D. immitis* isolates, and at different laboratories. Mice did not display any clinical signs associated with infection for up to 4 weeks. Developing larvae were found in subcutaneous and muscle fascia tissues, which is the natural site of this stage of heartworm in dogs. Compared with *in vitro-*propagated larvae at day 14, *in vivo-*derived larvae had completed the L4 molt, were significantly larger, and contained expanded *Wolbachia* endobacteria titres. We established an *ex vivo* L4 paralytic screening system whereby assays with moxidectin or levamisole highlighted discrepancies in relative drug sensitivities in comparison with *in vitro-*reared L4 *D. immitis*. We demonstrated effective depletion of *Wolbachia* by 70%−90% in *D. immitis* L4 following 2- to 7-day oral *in vivo* exposures of NSG- or NXG-infected mice with doxycycline or the rapid-acting investigational drug, AWZ1066S. We validated NSG and NXG *D. immitis* mouse models as a filaricide screen by *in vivo* treatments with single injections of moxidectin, which mediated a 60%−88% reduction in L4 larvae at 14–28 days.

**Discussion:**

Future adoption of these mouse models will benefit end-user laboratories conducting research and development of novel heartworm preventatives via increased access, rapid turnaround, and reduced costs and may simultaneously decrease the need for experimental cat or dog use.

## Introduction

*Dirofilaria immitis* is a major veterinary filarial nematode causing chronic heartworm disease (HWD) in dogs. Dirofilariasis is spread primarily by mosquito species of the *Culicidae* family, including the invasive tiger mosquito, *Aedes albopictus* (Cancrini and Kramer, 2001). HWD develops following the establishment of adult nematodes in the right chambers of the heart-associated vessels following larval migrations in subcutaneous and muscle tissues. Adult infections can persist in the heart for >5 years (McCall et al., 2008). Pathology is chronic-progressive, associated with enlargement and hyper-proliferation of endocardium and physical blockage of adult worms in the pulmonary artery contributing to vessel narrowing, hypertension, and ultimately heart failure (Simón et al., 2012). *Dirofilaria immitis* causes a more acute immunopathology in cats where the arrival of immature worms often triggers an overt inflammatory reaction in the lungs leading to heartworm-associated respiratory disease (McCall et al., 2008). Both cats and dogs are at risk of acute, fatal thromboembolisms when dead adult worms lodge in pulmonary vasculature (Simón et al., 2012). *Dirofilaria* spp. can also cause abbreviated zoonotic infections in humans, whereby the arrested development of immature adults can cause subcutaneous nodules and lung parenchyma disease (Reddy, 2013). *Dirofilaria repens* is the most widely reported dirofilarial zoonosis, noted to be increasing across Europe, Asia, and Sri Lanka, although, *D. immitis, Dirofilaria striata, Dirofilaria tenuis, Dirofilaria ursi*, and *Dirofilaria spectans* also infect humans (Litster and Atwell, 2008). In 2012, 48,000 dogs tested positive for heartworm in the United States (US), and in 2016, over one million pets were estimated to carry the disease.^1^ Incidence of HWD in the US is increasing both within endemic areas and into erstwhile HW-free, westerly and northerly regions, including Canada (Simón et al., 2012). A similar epidemiological pattern of increased dirofilariae incidence has also been documented in the Mediterranean, which has spread into the northern latitudes of Central and Western Europe (Morchón et al., 2012; Genchi and Kramer, 2017).

Heartworm disease is controlled by preventative chemotherapy and curative treatment of diagnosed cases. Chemo-prophylaxis with macrocyclic lactones (ML), namely ivermectin, milbemycin oxime, moxidectin, and selamectin, is effective at targeting L3–L4 larvae during subcutaneous tissue development and before immature adults reach the pulmonary artery to establish pathological adult infection (Wolstenholme et al., 2015; Prichard and Geary, 2019). After more than 40 years of use in veterinary medicine, ML drug resistance is prevalent in veterinary nematode parasites, with several *D. immitis* isolates formally determined as resistant to ML, whereby timed experimental infections and accurate prophylactic dosing have failed to prevent the development of fecund adult HW infections (Prichard and Geary, 2019).

The only regulatory-approved cure available for HWD is the injectable, melarsomine dihydrochloride. However, issues with this therapy include lengthy treatment regimens requiring in-clinic administrations, potential steroid pre-treatment, exercise restriction, and the risk of severe adverse events. Melarsomine is unsafe for use in cats, with no alternative curative therapies currently approved. Alternative curative therapies include the use of moxidectin and doxycycline (“moxi-doxy”) (Jacobson and DiGangi, 2021) with the latter antibiotic validated as a curative drug targeting the filarial endosymbiont, *Wolbachia*, demonstrable in human filariasis clinical trials (Johnston et al., 2021). However, due to concerns with doxycycline use within veterinary applications, such as long treatment time frames, dysbiosis side effects, and antibiotic stewardship of a human essential medicine, the development of short-course narrow-spectrum anti-*Wolbachia* heartworm therapeutics, without general antibiotic properties, may offer a potential future alternative (Turner et al., 2020).

ML preventatives, costing typically between $266 and $329 a year for a pet's treatment in the US, represent a potential multi-billion dollar global market (Mwacalimba et al., 2021). Due to the emergent spread of *D. immitis* infections, the growing concerns of ML prophylactic failure in the US, and the current inadequacies of curative treatments, new therapeutic strategies are being intensively investigated.

Until recently, the only fully validated *in vivo* screens available for heartworm anti-infectives were laboratory-reared cats and dogs. Lymphopenic and type-2 immunodeficient mice have been developed and validated as *in vivo* and *ex vivo* drug screens for medically important filarial parasite genera: *Brugia, Onchocerca*, and *Loa* (Halliday et al., 2014; Pionnier et al., 2019; Johnston et al., 2021; Marriott et al., 2022). Advantages of immunodeficient mouse models for filariasis drug screening include increased throughput, ease of maintenance, potential for international commercial supply, standardization with murine pharmacology models, reduced costs, and, potentially, a reduction in the use of “specially protected,” highly sentient animal species (cats, dogs, and non-human primates). Considering these advantages, academic investigators and animal healthcare companies have latterly begun to research rodent infection models of *D. immitis*, including the application of immunodeficient mice as potential drug screens (Noack et al., 2021; Hess et al., 2023). Here, we demonstrate that multiple lymphopenic immunodeficient mouse strains with ablation of the interleukin-2/7 common gamma chain (γc) are susceptible to the initial tissue larval development phase of *D. immitis*. *In vivo* larvae are morphologically superior to *in vitro*-propagated larvae (including *Wolbachia* endobacteria content), and can be successfully utilized in a variety of drug screening applications for the evaluation of direct-acting preventatives and anti-*Wolbachia* therapeutics.

## Materials and methods

### Animals

Male NOD.SCIDγc^−/−^ (NSG; NOD.Cg-*Prkdc*^*scid*^ *Il2rg*^*tm*1*Wjl*^/SzJ) and BALB/c RAG2^−/−^γc^−/−^ (RAG2ɤc; C;129S4-*Rag2*^*tm*1.1*Flv*^ *Il2rg*^*tm*1.1*Flv*^/J) mice were purchased from Charles River, UK. Male NXG mice (NOD-*Prkdc*^*scid*^*-IL2rg*^*Tm*1^/Rj) were purchased from Janvier Labs, France. Mice were group housed under specific pathogen-free (SPF) conditions at the biomedical services unit (BSU), University of Liverpool, Liverpool, UK. Male NSG mice used at TRS laboratories were purchased from The Jackson Laboratory, US, and group housed within filter-top cages. Mice were aged 5–7 weeks and weighed 21–32 g at the start of experiments. Animals had continuous access to fresh sterile food and water throughout experiments. Weight was monitored twice weekly and welfare behavior monitored daily. Study protocols were approved in the UK by LSTM & University of Liverpool Animal Welfare and Ethics Review Boards and licensed by The UK Home Office Animals in Science Regulation Unit. In the US, studies were approved by the TRS Institutional Animal Care and Use Committee.

### *Dirofilaria immitis* parasite production

Missouri isolate (MO) *D. immitis* microfilariae in dog blood (NR-48907, provided by the NIH/NIAID Filariasis Research Reagent Resource Center, FR3, for distribution through BEI Resources) were fed to female *Aedes aegypti* mosquitoes (Liverpool strain) at a density of 5,000 mf/ml through an artificial membrane feeder (Hemotek, UK). Blood-fed mosquitoes were reared for 15 days with daily sugar-water feeding to allow development to the L3 stage. At day 15, *Di*L3 were collected from infected mosquitoes by crushing and concentration using a Baermann apparatus and Roswell Park Memorial Institute (RPMI) 1640 with 1% penicillin–streptomycin (both Sigma-Aldrich, UK). For validation studies at TRS Labs, US, an in-house Georgia III (GAIII) isolate of *D. immitis* was utilized. *Dirofilaria immitis* mf were used to infect female *A. aegypti* mosquitoes (Liverpool strain) in dog blood using a glass feeder at a density of 1,000–2,500 mf/ml. At day 14, *Di*L3 were collected from infected mosquitoes using crushing and straining with RPMI 1640 and 1% penicillin–streptomycin.

### *Dirofilaria immitis* experimental infections

Highly motile infectious stage larvae (*Di*L3) retrieved from mosquitoes were washed in RPMI 1640 with 1% penicillin–streptomycin and 1% amphotericin B (Sigma-Aldrich, UK) and injected subcutaneously into the flank of male NSG, NXG, or RAG2ɤc mice at a density of 200 *Di*L3 per mouse. Cohorts of mice also received a single intraperitoneal injection of 2 mg methylprednisolone acetate (MPA; Sigma-Aldrich, UK) immediately prior to infection and after 1-week post-infection. Mice were humanely culled between 14 and 28 days post-infection. To retrieve parasites, skins were removed and subcutaneous tissue was scarified with a sharp scalpel blade. Muscle tissues were similarly scarified. Visceral organs were dissected and viscera, skin (pellet side-up), muscle tissues, and carcass were soaked in warm Eagle's minimum essential media (EMEM; Sigma-Aldrich, UK) with 1% penicillin–streptomycin and 1% amphotericin B for 2 h to allow active larvae to migrate from the tissues. Skin, muscle, and carcasses were incubated for a further 24-h period allowing residual larvae to migrate out of tissues.

### *In vitro* larval cultures

Madin-Darby Canine Kidney (MDCK) cells and rhesus monkey kidney epithelial (LLCMK2) cells were passaged in T-75 flasks in EMEM with 10% fetal bovine serum (FBS), 1% penicillin–streptomycin, 1% amphotericin B, and 1% non-essential amino acid solution (NEAAS; Sigma-Aldrich, UK). Cells were seeded onto 12-well plates to reach confluent monolayers 24–48 h prior to parasite addition. For parasite cultures, washed MO *Di*L3 from mosquitoes were plated onto cell monolayers, or the cell-free media (EMEM) control at a density of 10–20 iL3 per well with 4 ml media. Larvae were monitored over a 35-day time point for survival and motility and at 14 days post-culture to evaluate development, length, and *Wolbachia* titres.

### *In vitro* and *ex vivo* drug screening assays

MO *Di*L3 larvae were transferred onto MDCK monolayers and allowed to develop to 14- (early-mid L4) or 28 (mid-late L4)-day-old larvae. For comparative *ex vivo* assays, L4 stage larvae were recovered from male NSG mice 14 days post-infection and washed in sterile EMEM prior to the addition of drugs. All larval stages were plated into 12-well plates at densities of 3–5 larvae per well per drug concentration in 4 ml of EMEM with 10% FBS, 1% penicillin–streptomycin, 1% NEAAS, and 1% amphotericin B for drug screening assays. Moxidectin (Sigma-Aldrich, UK) was solubilised in phosphate-buffered saline (PBS, Fisher Scientific), and 10-fold serial dilutions ranging from 0.0001 to 100 μM were prepared in EMEM with 1% penicillin–streptomycin, 1% NEAAS, and 1% amphotericin B. Vehicle controls were included using the equivalent percentage PBS added to the cultures. Assays were incubated for 6 days in which larvae were continuously exposed to the drug at 37°C, 5% CO_2_ and scored daily for motility and survival.

### *In vivo* drug screening validation

Paired groups of 1–5 male NSG mice were subcutaneously inoculated with 200 *Di*L3 into the right flank on day 0. They were then randomized into treatment groups with a single subcutaneous dose of moxidectin prepared at 2.5 mg/kg in saline, or a saline-only control, in the nape of the neck on day 1. Mice were monitored daily for weight change and culled at day 14 to evaluate efficacy based on parasite recoveries. Alternatively, immediately following infection, groups of 4–6 mice were randomized into a 7-day oral regimen of doxycycline at 50 mg/kg prepared in ddH_2_O followed by a 7-day washout period, and a 2-day oral bi-daily regime of AWZ1066S prepared in standard suspension vehicle (SSV; PEG300/propylene glycol/H_2_O (55/25/20), or matching vehicle controls. Mice were monitored daily (weight and welfare) and culled between days 14 and 28 post-infection to evaluate parasitology, L4 length, and/or *Wolbachia* depletion using qPCR.

### *Wolbachia* titer analysis

To assess *Wolbachia* titres across different developmental time points (2, 3, and 4 weeks post-infection), to compare *in vitro*-reared larvae in parallel to *in vivo* reared controls, and to investigate drug activity against *Wolbachia*, individual larvae were taken, and their DNA was extracted using previously published methods (Halliday et al., 2014). *Wolbachia* single copy *Wolbachia surface protein* (*wsp*) gene quantification was undertaken by qPCR using the following primer pair: F-TTGGTATTGGTGTTGGCGCA and R-AGCCAAAATAGCGAGCTCCA, under conditions used to determine *Brugia malayi wsp* copy numbers (Halliday et al., 2014).

### Fluorescence *in situ* hybridization

Fluorescence *in situ* hybridization (FISH) was used for detecting *Wolbachia in Di*L3 and L4 larvae using two different DNA probes specific for *Wolbachia* 16S rRNA: W1—/5ATTO590N/AATCCGGC-GARCCGACCC and W2—/5ATTO590N/CTTCTGTGAGTACCGTCATTATC, as previously described by Walker et al. (2021). L3 and L4 larvae were stored in 50% ethanol at room temperature until further processing. For FISH staining, frozen larvae were fixed using 4% paraformaldehyde (PFA) and incubated with 10 μg/ml pepsin for 10 min at 37°C. After a thorough wash using PBS, the samples were hybridized overnight in hybridization buffer with probes (or without probes for negative controls). Hybridisation buffer consisted of 50% formamide, 5 × SCC, 0.1 M dithiothreitol (DTT), 200 g/L dextran sulfate, 250 mg/L poly(A), 250 mg/L salmon sperm DNA, 250 mg/L tRNA, and 0.5 × Denhardt's solution. Larvae were then washed twice in 1 × SSC and 0.1 × SSC 10 Mm DDT before mounting with VECTASHIELD antifade mounting medium containing DAPI (4′,6-diamindio-2-phenylinole;Vector laboratories). L4 larvae were visualized using bright-field microscopy for length measurements, calculated using Fiji (ImageJ), USA. FISH-stained larvae were imaged using a Zeiss laser scanning confocal microscope, and changes in larval morphology were visualized using bright-field and DAPI nuclear staining.

### Statistical analysis

Continuous data were tested for normality using the D'Agostino & Pearson omnibus Shapiro–Wilk normality tests. In case the data were skewed, non-parametric analyses were used to compare statistical differences between groups using Dunn's *post hoc* tests. In case the data passed the normality tests, Tukey's *post hoc* tests were applied. Categorical data were analyzed using Fisher's exact tests. Survival of larvae in culture (frequency motile vs. immotile) was evaluated using the log-rank (Mantel–Cox) test. Moxidectin/levamisole IC_50_ values were derived from the percentage of immotile larvae per drug concentration on day 6 of the assay. Non-linear curves were generated using the three-parameter least squares fit with [IC_50_] calculated. All the tests were performed using GraphPad Prism 9.1.2 software. Significance is indicated at or below alpha = 0.05.

## Results

### Selection of a susceptible mouse model of tissue-phase heartworm infection

**Figure 1:**
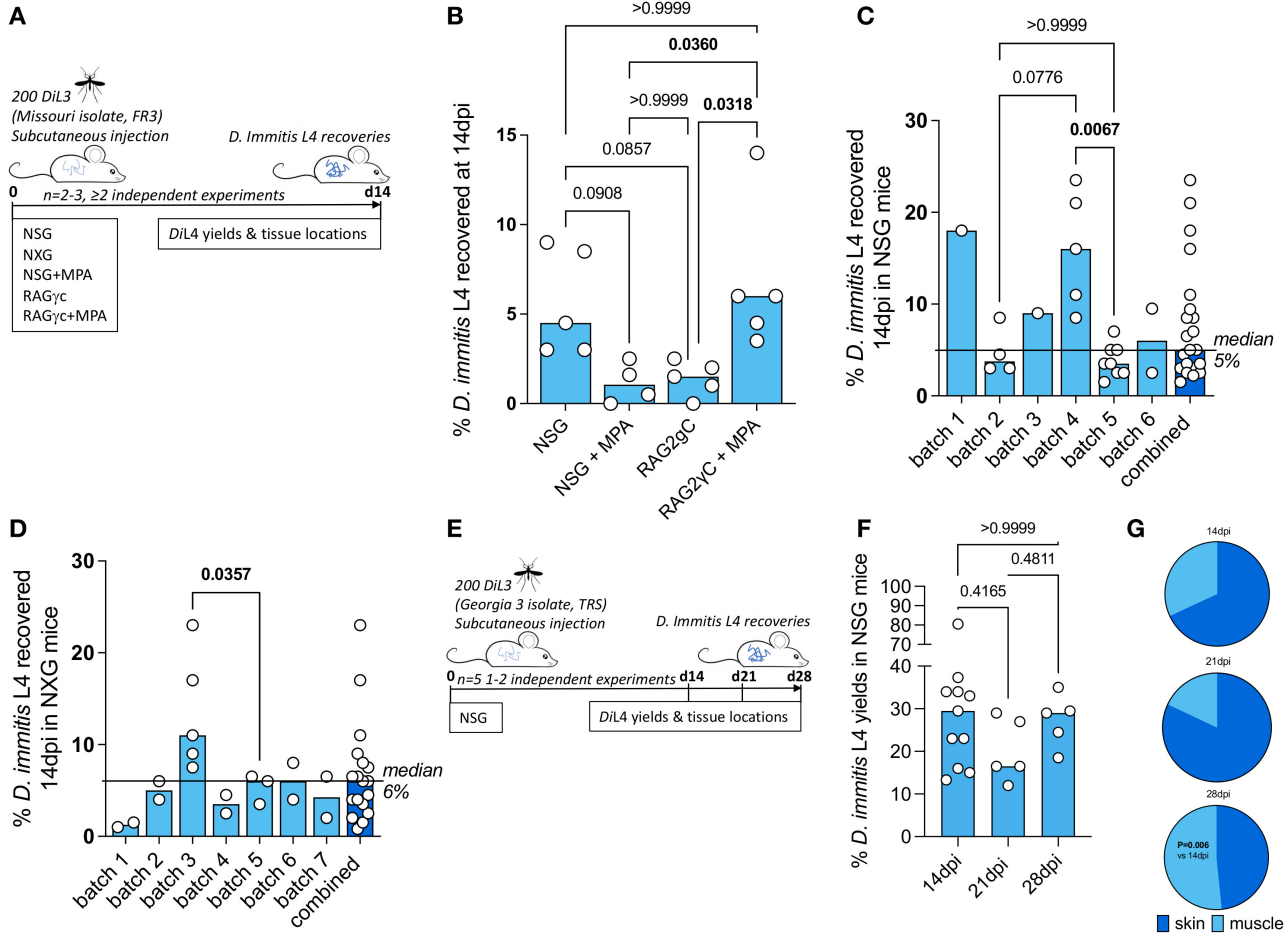
Susceptibility of compound immunodeficient mouse strains to *Dirofilaria immitis*. Experimental schematic using Missouri (MO) isolate *D. immitis* **(A)**. Percentage recovery of initial infectious load of *D. immitis* L4 at 14 days post-infection in indicated immunodeficient mice **(B–D)**. Experimental schematic using Georgia (GA)-III isolate *D. immitis* **(E)**. Percentage recovery of initial infectious load **(F)** and tissue distributions **(G)** of *D. immitis* L4 at indicated time points in NSG mice. Bars indicate median values with individual mouse data plotted. Significant differences were assessed by Kruskal–Wallis one-way ANOVA with Dunn's multiple comparison's tests except **(G)** where the difference in proportions was tested by Fisher's exact test. Significant differences (*P*-values < 0.05) are indicated in bold. Data are combined from two or more independent experiments **(B–D, F)** or individual experiments **(C, D, F)** with between 1 and 5 mice per group and per batch of L3 inoculated.

NSG and RAG2γc mouse strains were initially selected to investigate permissiveness to *D. immitis* tissue-phase larval infections, based on our previous success in establishing long-term infections of the related filarial species, *Brugia malayi, Loa loa*, and *Onchocerca ochengi*, utilizing lymphopenic immunodeficient mice (Halliday et al., 2014; Pionnier et al., 2019). In these models, the additional knockout of the interleukin-2/7 common gamma chain within lymphopenic mice is essential for susceptibility to *L. loa* adult development in subcutaneous tissues and bolsters both *B. malayi* and *O. ochengi* adult infections within the peritoneal cavity (Pionnier et al., 2022). We also trialed methylprednisolone acetate (MPA) administrations to evaluate whether steroid suppression of residual innate immune responses could increase survival and yields of *D. immitis* larvae *in vivo*, as has been reported for experimental *Strongyloides stercoralis* infections (Patton et al., 2018). Initially, we used a Missouri (MO) isolate of *D. immitis* (NR-48907, provided by the NIH/NIAID Filariasis Research Reagent Resource Center, FR3, for distribution through BEI Resources). Infectious L3 were isolated 15 days after membrane feeding of *D. immitis* mf in dog blood to *A. aegypti* at LSTM, and infection experiments were undertaken at the University of Liverpool, UK (Figure 1A). Following inoculations of 200 L3 under the skin, at 14 days post-infection, we successfully recovered *D. immitis* parasites from subcutaneous and muscle fascia tissues in all (5/5) NSG and RAG2γc + MPA infected mice (Figure 1B). Multiple tissues were dissected to locate parasites (heart, lungs, peritoneal cavity, gastrointestinal tract, liver, and spleen), but no evidence of infection was found in these ectopic locations. Infection success was lower in NSG + MPA (3/4 mice) and RAG2γc (4/5) mouse groups. Yields significantly varied between groups with RAG2γc + MPA mice yielding higher numbers of *D. immitis* developing larvae (L4) compared with either RAG2γc or NSG + MPA groups (Kruskal–Wallis one-way ANOVA *P* = 0.0033 and Dunn's *post hoc* tests *P* < 0.05). Median recovery rates were similar between NSG and RAG2γc + MPA groups (median recovery 9 vs. 12%, ns).

Due to the simpler infection regimen in NSG mice, the international commercial availability of the model, the potential for further humanization, and the potential to avoid welfare or drug-drug interactions arising due to long-term MPA administrations, we selected this immunodeficient mouse model for extensive characterization. We evaluated the infection success and yields of *D. immitis* L4 across multiple independent experiments utilizing different batches of MO isolate *D. immitis* shipped from the US to the UK as mf in dog blood and passaged to the infectious L3 stage in *A. aegypti* mosquitoes (Figure 1C). In six independent experiments, using a total of 21 mice, we were reproducibly able to recover *D. immitis* larvae at 14 dpi (21/21 mice) with a 5% median yield of the initial 200 L3 infectious inoculate (range 1.5%−23.5%). In experiments where >2 mice were infected, we compared yields between batches and determined that batch-to-batch variability of the initial L3 infectious inoculate significantly influenced the yields of larvae recovered at 14 dpi in NSG mice (Kruskal–Wallis one-way ANOVA *P* = 0.0017 and Dunn's *post hoc* tests *P* < 0.01). We then measured recoveries utilizing NXG mice; a similar severe combined immunodeficient mouse line on the non-obese diabetic strain background with additional γc ablation developed independently and recently commercialized by Janvier Laboratories^2^ (Figure 1D). As with the NSG mouse line, in seven independent experiments, with a total of 18 experimental mouse infections (run at LSTM/University of Liverpool), we consistently recovered developing larvae at 14 dpi (18/18 NXG mice) with a 6% median proportion of the initial 200 L3 infectious inoculate (range 1%−23%). Similar to NSG infections, in individual experiments where >2 NXG mice were available for analysis, batch-to-batch variability of the initial L3 infectious inoculate significantly influenced the yields of larvae recovered at 14 dpi (Mann–Whitney *P* < 0.05). We then repeated experiments with NSG mice in TRS Labs (Georgia, USA), accessing an in-house parasite life cycle and using a unique “Georgia III” (GAIII) isolate of *D. immitis*. We infected batches of five mice and evaluated yields of L4 at 14, 21, or 28 days post-inoculation with 200 L3 (Figure 1E). All mice, irrespective of time point post-inoculation, yielded GAIII *D. immitis* developing larvae. Yields were six-fold higher on average than those derived at the LSTM laboratory at 14 days post-infection (median = 29.5%, range 13.3%−80.5%, Figure 1F). Yields did not significantly deviate between 14, 21, and 28 days post-infection (Figure 1F). However, the distribution of larvae in mouse tissues changed between 14 and 28 days post-infection, with relatively more larvae recovered in muscle tissues by 28 dpi (*P* < 0.01, Fisher's exact test, Figure 1G). These experiments demonstrate that lymphopenic mice with additional IL-2 gamma chain deficiency are susceptible to *D. immitis* tissue-stage infection with reproducible success using different isolates of heartworm in independent laboratories and when shipping larvae internationally between sites (details of which are summarized in Supplementary Table 1). Our time-course data indicate that tissue-phase heartworm larvae persist without significant decline in yields within NSG mice whilst initiating their natural migratory route through subcutaneous and muscle tissues over the first 28 days of infection.

### Mouse-derived developing larvae demonstrate superior morphogenesis, *Wolbachia* content, and reduced drug assay sensitivities compared with *in vitro* cultured *D. immitis*

**Figure 2:**
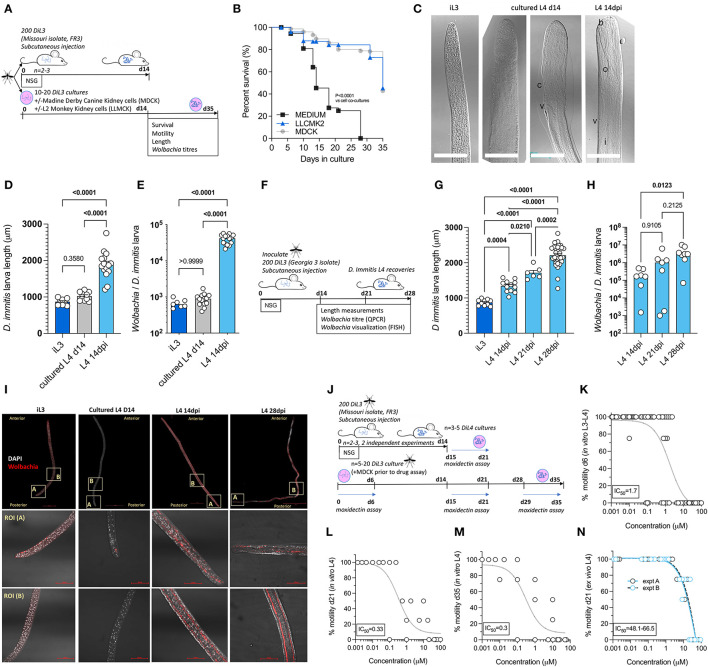
Comparative morphogenesis, *Wolbachia* expansions, and drug assay sensitivities of mouse-derived larvae compared with *in vitro* cultured *Dirofilaria immitis*. Experimental schematic using MO isolate *D. immitis* L3 **(A)**. Survival analysis of cultured larvae in indicated conditions **(B)** representative photomicrographs of iL3, d14 L4 cultures or L4 recovered from NSG mice at 14 dpi [**(C)**; b, buccal cavity; c, cuticle; i, intestine; o, esophagus; v, vulva, and scale bars = 50 μM]. Length **(D)** and *Wolbachia* content **(E)** of iL3, d14 L4 cultures or L4 recovered from NSG mice at 14 dpi. Experimental schematic using GAIII isolate *D. immitis* L3 **(F)**. Length **(G)** and *Wolbachia* content **(H)** of NSG mouse-derived L4 larvae at indicated time points. Representative FISH photomicrographs of iL3, d14 L4 cultures or L4 recovered from NSG mice at 14 or 28 dpi (*Wolbachia* 16SrRNA red, DAPI gray, scale bars = 50 μM) **(I)**. Experimental schematic of drug assays utilizing MO isolate *D. immitis* cultures or *ex vivo* larvae from NSG mice **(J)**. Moxidectin concentration *D. immitis* motility inhibition analysis after 6-day exposures when using cultured *D. immitis* for periods between 0 and 6 days L3/L4 **(K)**, 15–21 days L4 **(L)**, 28–35 days L4 **(M)**, or 15–21 days L4 derived from 14 dpi NSG mice **(N)**. In **(K–N)**, non-linear curves are three-parameter least squares fit with [IC_50_] calculated in Prism 9.1.2. Bars represent mean ± SEM **(D, G)** or median **(E, H)** values with individual larva data plotted. Significant differences were determined by the Mantel–Cox log-rank tests **(B)**. One-way ANOVA with Tukey's multiple comparisons tests **(D, G)** or Kruskal–Wallis with Dunn's multiple comparisons tests **(E, H)**. Significant differences (*P*-values < 0.05) are indicated in bold. Data are one individual experiment except **(N)** which is two independent experiments.

Mosquito-derived infectious L3 larvae are traditionally utilized in serum-supplemented 37°C mammalian cultures to induce molting and morphogenesis into fourth-stage developing larvae (Lok et al., 1984; Devaney, 1985; Abraham et al., 1987). This technique has been utilized to study *D. immitis* larval biology and for applied applications such as biomarker and preventative drug discovery (Long et al., 2020; Hübner et al., 2021; Tritten et al., 2021). The survival of various filarial parasite life cycle stages can be extended when utilizing co-cultures with mammalian “feeder cell” monolayers or trans-well compartments (Townson et al., 1986; Evans et al., 2016; Zofou et al., 2018; Njouendou et al., 2019; Gandjui et al., 2021; Marriott et al., 2022). We, therefore, compared the survival and motility of MO isolate *D. immitis* larvae between cell-free and LL-MCK2 (monkey) or MDCK (dog) kidney cell co-cultures in 10% calf-serum cultures (Figure 2A). The 50% survival time of cell-free cultures was day 18, and subsequently, all larvae had died by day 28 in culture (Figure 2B). Conversely, co-cultures with both LL-MCK2 and MDCK cells significantly increased survival, whereby >80% of *D. immitis* larvae were viable up to day 28 (*P* < 0.0001, Mantel–Cox survival analysis). We noted a reduction in motility in all larval cultures after the first week in culture, which persisted to end point, apart from MDCK co-cultures which returned to full motility by day 32 in culture (Supplementary Figure 1). Selecting MDCK co-cultures as supportive of long-term larval motility and survival, we directly compared morphogenesis, growth, and *Wolbachia* endobacteria expansions between *in vitro-*propagated MO *D. immitis* larvae and MO larvae derived from NSG mouse infections at the 14-day time point (Figure 2A). Both *in vitro-* and *in vivo-*derived d14 *D. immitis* larvae displayed the blunted and widened anterior extremities characteristic of the L4 developmental stage (Orihel, 1961; Kotani and Powers, 1982), compared with the tapered, narrow anterior of filariform infectious L3 (Figure 2C). However, anterior morphogenesis was partially arrested *in vitro* compared with NSG mouse-derived larvae (Figure 2C). In the dog, larvae complete the L3–L4 molt rapidly, the vast majority by 3 days post-infection (Lichtenfels et al., 1985). In our cultures, ~50% of the day 14 L4 had completed molting, with cuticle casts evident in the culture media. The other ~50% of *in vitro* cultured larvae displayed partial molting of the third-stage cuticle (Figure 2C). There were obvious microscopic degenerative features of the *in vitro* larvae by day 14 compared with *in vivo* larvae, including malformed cuticle, hypodermis, buccal cavity, esophagus, and intestine (Figure 2C). Despite their high survival rate and continued motility, 14-day-old *in vitro*-propagated larvae were also significantly stunted compared with larvae derived from NSG mice (mean = 1020 vs. 1880 μM, one-way ANOVA *F* = 57.7, *P* < 0.0001, Tukey's multiple comparisons test) and had not grown significantly in comparison with the L3 infectious stage (mean = 870 μm; Figure 2D). *Wolbachia* titer analysis using qPCR further highlighted disparities between *in vitro* and *in vivo* reared larvae (Figure 2E). The MO *D. immitis in vivo* larvae had undergone a significant, 66-fold average *Wolbachia* expansion during the 14-day NSG mouse (Figure 2E) infection in comparison with iL3 (median = 4.2 × 10^4^ vs 6.2 × 10^2^ *Wolbachia*/larva, Kruskal–Wallis one-way ANOVA 26.4, *P* < 0.0001 Dunn's multiple comparisons test), whereas MO larvae cultured for 14 days *in vitro* had failed to expand *Wolbachia* content (median = 8.7 × 10^2^ *Wolbachia*/larvae).

Utilizing GAIII *D. immitis*, we further examined length and *Wolbachia* expansions between day 14 and day 28 post-infection in NSG mice (Figure 2F). GAIII L4 continued to grow in length between day 14, day 21, and day 28 post-infection in NSG mice (Figure 2G; means = 1,335, 1,713, and 2,211 μm, respectively, one-way ANOVA *F* = 87.4, *P* < 0.05–*P* < 0.0001, Tukey's multiple comparisons tests). Similarly, *Wolbachia* titres continued to expand within the NSG-derived GAIII *D. immitis* larvae (Figure 2H) with significant differences evident between day 14 and day 28 (median = 1.7 × 10^5^ vs. 3.1 × 10^6^ *Wolbachia/*larva, Kruskal–Wallis statistic = 8.5, *P* < 0.05 Dunn's multiple comparisons test). We corroborated qPCR *Wolbachia* data, visualizing that time-dependent *Wolbachia* multiplication was occurring within the hypodermal chord cell syncytia from a posterior to anterior direction in mouse-derived, but not *in vitro* cultured, *D. immitis* L4 specimens, utilizing fluorescent *in situ* hybridization (FISH) of *Wolbachia* 16S rRNA and confocal microscopy (Figure 2I). We then examined the *in vitro* vs. *ex vivo* paralytic susceptibilities of MO isolate *D. immitis* L4 following 6-day exposures to the standard preventative drug, moxidectin, using cultured L3-L4 larvae at 0–6 days, 15–21 days, or 28–35 days compared with NSG mouse L4 larvae isolated at 14 dpi and exposed to drug *ex vivo* between 15–21 days in matching culture conditions (Figure 2J). The IC_50_ concentrations inhibiting motility of *D. immitis* were 1.7 μM for 0–6 days L3–L4 larvae (Figure 2K). Sensitivity to moxidectin had increased in day 14–day 35 larvae with IC_50_ ranging between 300 and 330 nM (Figures 2L, M). In comparison, *ex vivo* larvae derived from mice were relatively insensitive to the *in vitro* paralytic activity of moxidectin with IC_50_ ranging between 48 and 66 μM (Figure 2N). This equated to a >28-fold decrease in moxidectin susceptibility compared with *D. immitis* L3–L4 cultures and >140-fold decreased sensitivity compared with long-term L4 cultures. We further examined relative paralytic susceptibilities of *D. immitis* MO *in vitro* vs. *ex vivo* L4 to 6-day exposures of the anthelmintic, levamisole, commencing at 15 days after iL3 culture/infection (Supplementary Figure 2). Whilst *in vitro* larvae were susceptible to high doses of levamisole (IC_50_ 13.2 μM), *ex vivo* L4 maintained full motility for 6 days in the presence of the top dose of the drug (100 μM). These data demonstrate the developmental superiority of *D. immitis* larvae derived from the subcutaneous and muscle tissues of NSG mice compared with standard *in vitro* cultures, reflected in a lowered sensitivity to moxidectin and levamisole when used in *ex vivo* drug titration assays.

### *Dirofilaria immitis* NSG or NXG mouse infections can be used to evaluate anti-*Wolbachia* drugs

**Figure 3:**
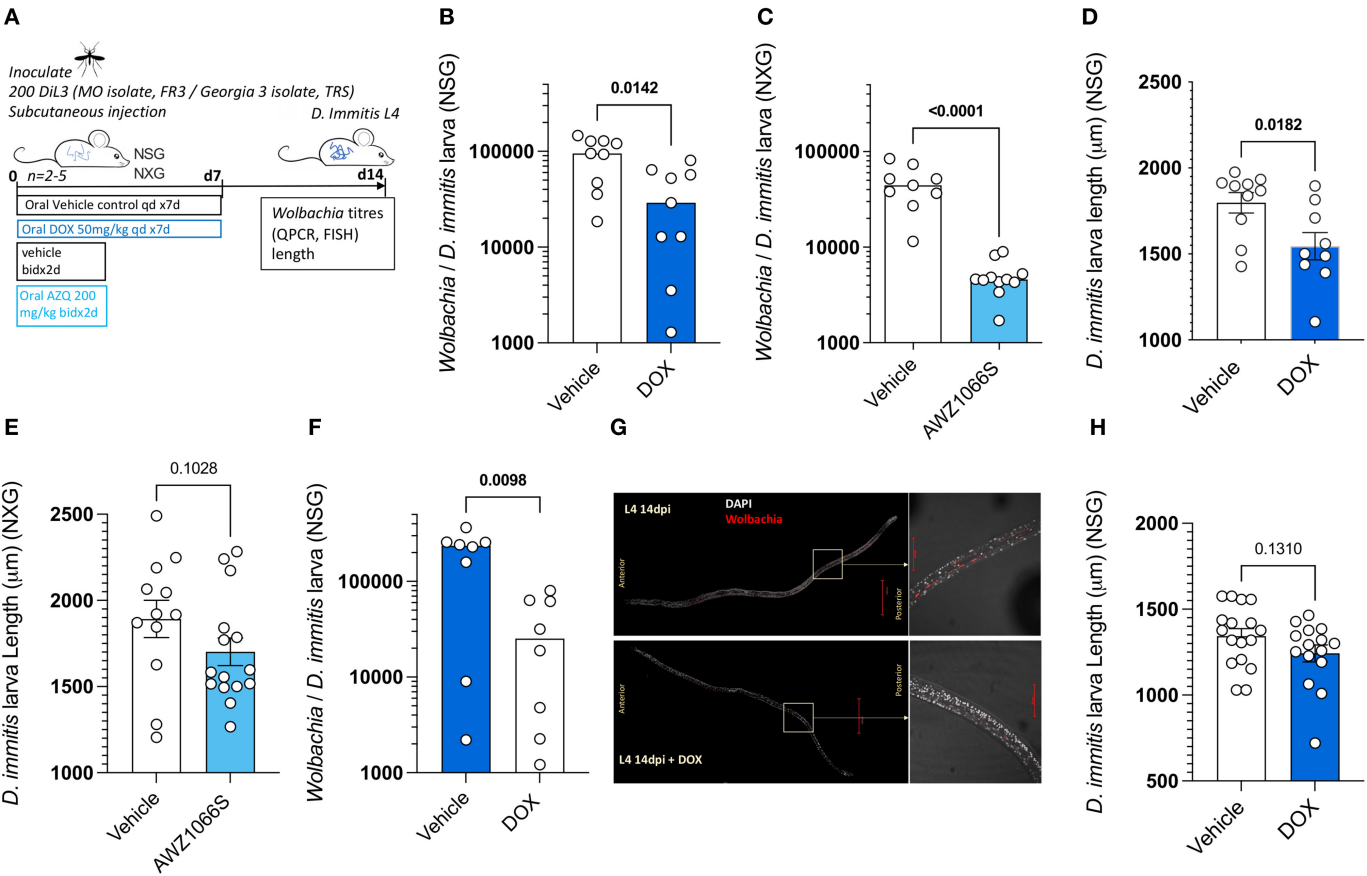
Validation of *Dirofilaria immitis* NSG and NXG mouse models as *in vivo* anti-*Wolbachia* drug screening systems. Experimental schematic using MO or GAIII isolates of *D. immitis* L3 **(A)**. *Wolbachia* loads determined by qPCR on day 14 MO *D. immitis* larvae exposed to doxycycline **(B)** or AWZ1066S **(C)**. Length changes in day 14 MO *D. immitis* larvae exposed to doxycycline **(D)** or AWZ1066S **(E)**. *Wolbachia* loads determined by qPCR on day 14 GAIII *D. immitis* larvae exposed to doxycycline **(F)**. Visualization of *Wolbachia* depletion in hypodermis of day 14 GAIII *D. immitis* larvae exposed to doxycycline by FISH **(G)**. Length changes in day 14 GAIII *D. immitis* larvae exposed to doxycycline **(H)**. Bars represent median **(B, C, F)** or mean ± SEM **(D, E, H)** values with individual larva data plotted. Significant differences were determined by Mann–Whitney tests **(B, C, F)** or unpaired Student's *t*-tests **(D, E, H)**. Significant differences (*P*-values < 0.05) are indicated in bold. Data are one individual experiment with larvae derived from 2 to 5 NSG **(B, D, F, G)** or NXG **(C, E)** mice per group.

Because we established rapid *Wolbachia* expansions occur during the L4 tissue development phase of *D. immitis* following infections of NSG or NXG mice, we next investigated the validity of these models as anti-*Wolbachia* drug screens. We initially infected batches of 4–6 NSG mice with MO isolate *D. immitis* iL3 and randomized animals into 7-day 50 mg/kg oral treatment with doxycycline or matching vehicle controls, commencing at infection with a further 7-day washout period to 14 dpi (Figure 3A). We selected this regimen and timing of dose based on proven significant depletion of *B. malayi* L3-L4 *Wolbachia in vivo* in a SCID mouse model (Jacobs et al., 2019). We subsequently randomized four NXG-infected mice into a 2-day bi-daily 200 mg/kg treatment of our fast-acting anti-*Wolbachia* azaquinazoline clinical candidate, AWZ1066S (Hong et al., 2019) or vehicle control, to compare relative anti-*Wolbachia* activity. Doxycycline treatment mediated a 70% median reduction in *Wolbachia* titres in day 14 MO *D. immitis* larvae when compared against vehicle control levels (0.29 × 10^4^ vs. 9.5 × 10^4^ *Wolbachia*/larva, Mann–Whitney test *P* = 0.014, Figure 3B). The short-course AWZ1066S 2-day oral treatment mediated a more profound 90% median efficacy in depletion of *Wolbachia* from day 14 MO *D. immitis* larvae (0.49 × 10^4^ vs. 4.5 × 10^4−^ *Wolbachia*/larva, Mann–Whitney test *P* < 0.0001, Figure 3C). The effect of *Wolbachia* depletion via doxycycline and AWZ1066S on larval growth was also evaluated (Figures 3D, E). We found that depletions of *Wolbachia* by 7-day doxycycline or 2-day AWZ1066S were associated with a 15.9 and 15.3% mean stunting effect on 14-day MO *D. immitis* larvae, which was significant for doxycycline treatment (Student's *t*-test, *P* = 0.0182). We repeated the validation of the *D. immitis* NSG mouse model as an anti-*Wolbachia* drug screening system in an independent laboratory, utilizing GAIII isolate *D. immitis* (Figure 3A). In this dosing study, the 7-day oral regimen of doxycycline mediated a significant, 89% median depletion of *Wolbachia* in day 14 GAIII larvae (0.25 × 10^5^ vs. 2.4 × 10^5^ *Wolbachia*/larva, Mann–Whitney test, *P* = 0.0098, Figure 3F). We corroborated the clearance of *Wolbachia* from posterior hypodermal chord cells by FISH staining (Figure 3G). Stunting of GAIII *D. immitis* larvae was also apparent following 7-day doxycycline exposures in NSG mice (mean reduction in length 30%, Figure 3H). Together, these data demonstrate the utility of the *D. immitis* tissue-phase NSG or NXG mouse models to screen for the efficacy of oral anti-*Wolbachia* regimens *in vivo*.

### *Dirofilaria immitis* NSG and NXG mouse infections can be used to evaluate preventative drug efficacy

**Figure 4:**
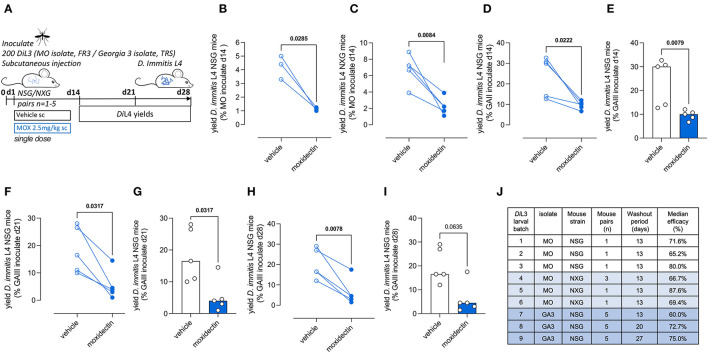
Validation of *Dirofilaria immitis* NSG and NXG mouse models as *in vivo* larvicidal drug screens. Experimental schematic using MO or GAIII isolates of *D. immitis* L3 **(A)**. Change in *D. immitis* larval recoveries at day 14 in randomized NSG **(B)** or NXG **(C)** mouse pairs inoculated with MO isolate *D. immitis* and treated with vehicle or 2.5mg/kg moxidectin via subcutaneous injection at day 1. Change in *D. immitis* larval recoveries in randomized NSG mouse matched pairs **(D, F, H)** or as independent groups of *n* = 5 **(E, G, I)** following inoculation with GAIII isolate *D. immitis* and treatment with vehicle or 2.5mg/kg moxidectin subcutaneous injection at day 1. Summary of median moxidectin efficacies derived from NSG or NXG mouse pairs inoculated with different batches of MO or GAIII L3 *D. immitis* larvae and treated with vehicle or moxidectin, evaluated at 14, 21, or 28 days **(J)**. Significant differences between matched pairs were determined by paired *t*-tests **(B–D, F, H)** or as independent groups by Mann–Whitney Tests **(E, G, I)**. Significant differences (*P*-values < 0.05) are indicated in bold. Data are pooled from three individual experiments **(B, C)** or one individual experiment **(D–I)**, derived from 1, 3, or 5 mouse pairs.

Moxidectin is a front-line ML preventative used in various oral, topical, or injectable formulations as monthly, biannual, or annual heartworm prophylaxis in dogs (Savadelis et al., 2022). We selected a single high-dose subcutaneous injection of moxidectin (2.5 mg/kg) for the evaluation of larvicidal efficacy in NSG or NXG mice (emulating route of delivery and dose of long-acting injectable formulations of moxidectin in dogs). Matched pairs of mice were infected with batches of 200 *D. immitis* MO or GAIII isolate larvae, and the next day these were randomized into vehicle control or moxidectin treatment (Figure 4A). After 14 days post-infection (13 days post-treatment), we recorded 65%−80% reductions in MO *D. immitis* L4 in NSG mice (*n* = 3 pairs, *P* = 0.03, paired *t-test*, Figure 4B). A similar range of larvicidal efficacy was evident in NXG mice 14 days after infection with MO isolate *D. immitis* and treatment with a single injection of moxidectin (range 46%−88% *n* = 6 pairs, *P* = 0.008, Figure 4C). When using the GAIII isolate of *D. immitis* for NSG infections, the level of moxidectin efficacy ranged between 29 and 73%, evaluated at 14 days post-infection (*P* = 0.022, *n* = 5 pairs, Figure 4D). The availability of groups of five mice infected with the same batch of L3 allowed for unpaired group testing, rather than matched pairs, whereby the significance of moxidectin efficacy was confirmed (*P* = 0.008, Mann–Whitney Test, Figure 4E). We examined extended washout periods after moxidectin single dosing in NSG mice infected with GAIII *D. immitis*. At 21 days post-infection, the range of moxidectin efficacy was 45%−94% (*P* = 0.037, *n* = 5 paired analysis and Mann–Whitney tests Figures 4F, G) whilst at 28 days post-infection, efficacy ranged between 35 and 91% (*P* = 0.0078, *n* = 5 paired analysis, *P* = 0.064, Mann–Whitney tests, Figures 4H, I). The median efficacy for all studies is shown in Figure 4J. In summary, a single injection of moxidectin delivered a median efficacy between 65 and 80% at 2 weeks in NSG/NXG mice infected with MO isolate, and 60%, 73%, and 75% efficacy at 2, 3, or 4 weeks in NSG infected with GAIII isolate (Figure 4J). All mice in the drug studies displayed typical behavior and gained weight over the 2- to 4-week period of infection and dosing (Supplementary Figure 3).

## Discussion

We have determined that ablation of both the B and T lymphocyte compartments and additional cytokine signaling via the IL-2/7 common gamma chain receptor in mice allows permissiveness to *D. immitis* tissue-phase larval development for at least the first 28 days of infection. This confirms the study of Hess et al. (2023) who recently reported the NSG strain as a *D. immitis* mouse model but also highlights that other commercially available mouse strains with similar deficiencies (NXG and BALB/c RAG2^−/−^γc^−/−^) are susceptible to *D. immitis* tissue-phase L4 infections. A polarized type-2 adaptive immune response with associated type-2 tissue macrophage activation leads to eosinophil entrapment and degranulation as the basis of immune-mediated filarial larvicidal activity in mice (Specht et al., 2006; Turner et al., 2018; Pionnier et al., 2020; Ehrens et al., 2021). However, experimental infections with *B. malayi, L. sigmodontis*, and *O. ochengi* in lymphopenic mouse strains (SCID/RAG2^−/−^) with additive γc gene ablations have illustrated bolstered chronic susceptibility (Layland et al., 2015; Pionnier et al., 2022), whilst in *L. loa* subcutaneous infections, only combination of lymphopenia and γc deficiency is sufficient to allow permissiveness to adult infections (Pionnier et al., 2019). Thus, an additional layer of innate immune resistance operates which can reduce or eliminate establishing larval filarial infections. In *B. malayi* infections, p46^+^ NK cells with an activated/memory phenotype and residual eosinophilia are implicated in the innate immune resistance to chronic infection in RAG2^−/−^ mice (Pionnier et al., 2022), whereas in *Litomosoides* infections, CD45^−^/TCRβ^−^/CD90.2^+^/Sca-1^+^/IL-33R^+^/GATA-3^+^ type-2 innate lymphoid cells (ILC2) are required for innate immune resistance to microfilarial blood infections (Reichwald et al., 2022). It remains to be determined which of these multiple innate and adaptive immune processes are operating to control the larval establishment of *D. immitis* in mice. Because we detected increases in *D. immitis* larval burdens in NOD.SCID- vs. BALB/c RAG2^−/−^-γc^−/−^ mice which could be improved by steroid treatments in the latter model, this may indicate residual innate immune differences between background strains. For instance, NOD mice are deficient in complement humoral immunity due to a 2-bp deletion in the haemolytic complement (Hc) gene, which encodes the C5 complement protein (Verma et al., 2017). Our models now afford an opportunity for reconstitution of innate or adaptive immune cell types and humoral immune components to dissect the mechanisms of immunity to *D. immitis* migrating larvae, as has recently been attempted for *L. sigmodontis* with CD4^+^ T cell transfers into RAG2^−/−^γc^−/−^ mice (Wiszniewsky et al., 2021). This application may be useful in determining minimally sufficient immune pathways necessary to mediate sterilizing immunity. Similarly, the new *D. immitis* mouse models may be valuable in evaluating the efficacy of neutralizing sub-unit vaccine target antibody responses (e.g. via passive transfer of purified specific antibodies or isolated B-cell clones adoptively transferred from immunocompetent NOD mice).

Our initial evaluations of immunodeficient mouse susceptibility utilized MO isolate *D. immitis* mf shipped from the FR3 repository, Athens, USA, to Liverpool, UK, before being reared to infectious stage L3s within Liverpool Strain *A. aegypti*. Following the selection of the NSG and NXG mouse lines for further evaluations, when we repeated experiments in an independent laboratory (TRS, Georgia) with NSG mice infected with an on-site *A. aegypti* generated GAIII isolate of *D. immitis*, the yields of L4 at 2 weeks were improved on average by ~six-fold. The latter yields were more in line with those achieved by Hess et al. (2023) (16%−29% recoveries), utilizing the FR3 MO isolate whereby L3s were generated locally. We currently do not know the factor or factors causing variable susceptibility between laboratories, but they could include the impact of international shipping of mf, disparities in L3 larval infectivity following passage in different colonies of mosquitoes, or variances in the maintenance of immunodeficient mice between facilities altering host microbiome and modifying host innate response to infection. Because we recorded significant batch-to-batch variability in L3 inoculations in terms of L4 recovered at 14 days, the quality of infectious stage L3 produced by mosquito colonies is likely to be a major influencing factor, and experiments should be carefully controlled to account for this potential source of batch variation.

We evaluated that *D. immitis* larval parasitism and development in immunodeficient mice accurately tract the natural course of infection in definitive hosts over the 1st month. All larvae were recovered from the subcutaneous tissues and muscle fascia, in line with previous observations of natural parasite locations in both ferret and dog infections of *D. immitis* at this time interval (Orihel, 1961; Supakorndej et al., 1994). We demonstrate that *in vivo* larvae complete cuticle molting and undergo 4th stage larval morphogenesis. L4 growth lengths in NSG or NXG mice were within the range of those prior documented in dog and ferret infections at matching point of infection, at 14–15 days (NSG = 1.2–2.8 mm, NXG = 1.2–2.5 mm, dog = 1.7–2.2 mm, ferret = 1.6–2.7 mm) (Orihel, 1961; Supakorndej et al., 1994). These lengths also emulate those recently reported (1.5–1.8 mm) after 14 days of infection of NSG mice by Hess et al. (2023).

We also demonstrate that *D. immitis* expand *Wolbachia* titres significantly during parasitism of NSG or NXG mice. From PCR analysis, we ascertain that *Wolbachia* are doubling approximately every 42 h for MO isolate to every 55 h for GAIII isolate over the first 14-day infection time-course of *D. immitis* L3–L4 larvae *in vivo*. This is the first record of early *Wolbachia* expansions in *D. immitis* developing larvae and is comparatively slower compared with the average doubling time (32 h) over the first 14 days of L3–L4 development *in vivo* for the human filariae, *B. malayi* (McGarry et al., 2004). The establishment of *D. immitis* mouse models now allows for tractable comparative endosymbiotic biology of this clade C nematode *Wolbachia* (also found in the causative agent of river blindness, *O. volvulus*) vs. the clade D *Wolbachia* of human lymphatic filariae, most commonly used in basic and applied nematode *Wolbachia* research.

Before the establishment of *D. immitis* immunodeficient mouse models, a ready source of *in vivo D. immitis* L4 propagations for onward “*ex vivo*” basic and translational research has been unavailable. Mosquito stage L3 can be induced to molt rapidly into the early L4 stage, with as much as 95% molting success, and survive for 3 weeks in calf serum–supplemented cultures (Abraham et al., 1987). We recapitulated this early L4 morphogenesis and improved L4 longevity to >1 month in culture if larvae were co-cultured with dog or monkey kidney cells. However, comparisons with *in vivo* reared larvae highlighted several defects in growth, incomplete morphogenesis, and, most strikingly, an almost complete failure to expand *Wolbachia* endosymbiont titres. Therefore, the failure of larvae to thrive *in vitro* may be linked with a deficit in *Wolbachia-*produced haem, riboflavin, nucleotides, or other biosynthetic pathways identified as relevant in the *Wolbachia–*nematode symbiosis (Lefoulon et al., 2020). Whether environmental cues are lacking *in vitro* for *Wolbachia* expansion is currently not known. Sub-optimal neo-glucogenesis in cultured *D. immitis* larvae could lower available carbon energy sources necessary for *Wolbachia* expansion (Voronin et al., 2019). Alternatively, because autophagic induction in filariae regulates *Wolbachia* populations residing within host vacuoles (Voronin et al., 2012), failure of *Wolbachia* growth may be the result of starvation/stress in culture-inducing autophagy. Certainly, filarial stress responses are demonstrably upregulated in *ex vivo* adult worm culture systems (Ballesteros et al., 2016).

We demonstrate that the use of sub-optimal L3/L4 grown *in vitro* for pharmacological screening leads to significantly >28-fold increased sensitivities to the paralytic activities of two nematodicidal agents, namely moxidectin and levamisole, compared with larvae of the same age derived from NSG or NXG mouse infections. Our data are currently limited to comparisons of two drugs, and more evaluations are required to determine how consistently *ex vivo* vs. *in vitro* larvae diverge in terms of drug sensitivity. However, our data suggest that a reliance on *in vitro* larvae may lead to artificial sensitivities to new preventatives in development and thus might lead to incorrect selection of candidates or dose levels for *in vivo* preclinical evaluations with consequences for incorrect cat and dog usage. The mouse models now afford a facile method of generating more physiologically relevant L4 larvae which should offer more accurate pharmacological assessments prior to a decision to advance into *in vivo* preclinical screening justifying protected animal use.

Our data determining the failure of *Wolbachia* to expand *in vitro* within *D. immitis* developing larvae preclude the use of cultured L3/L4 in evaluating the activities of novel anti-*Wolbachia* compounds. We thus demonstrate the utility of the NSG and NXG mouse models as an *in vivo* anti-*Wolbachia* drug screen. L4 larvae could be reproducibly depleted of *Wolbachia* using a 7-day regimen of the established anti-*Wolbachia* antibiotic, doxycycline, with confirmatory experiments run in an independent laboratory with a different *D. immitis* isolate. The levels of anti-*Wolbachia* efficacy we observed (70%−89%) are aligned to those measured against *B. malayi* day 14 larval *Wolbachia* following identical doxycycline regimen treatment of infected CB.17 SCID mice (76%) (Jacobs et al., 2019). Excitingly, we demonstrate that a 2-day *in vivo* treatment with the novel investigational azaquinazoline drug, AWZ1066S, is a rapid and profound *D. immitis Wolbachia* depleting agent with 90% efficacy achieved. This benchmark of 90% efficacy has been determined as clinically relevant in terms of sustained *Wolbachia* reductions and subsequent long-term anti-parasitic activities in human filariasis clinical trials (Johnston et al., 2021). The unique rapid activity of AWZ1066S has been previously determined through time-kill assays with *B. malayi Wolbachia*, whereby a near maximum kill rate can be achieved with 1-day exposure compared with 6 days for standard classes of antibiotics, including tetracyclines (Hong et al., 2019). Thus, azaquinazolines or other novel anti-*Wolbachia* chemistry with similar rapid killing activity, as identified in high-throughput industrial screening (Clare et al., 2019), might hold promise as new heartworm preventative or curative candidates and now can be triaged for activity utilizing our novel *D. immitis* mouse models.

We used a high single parenteral dose of moxidectin, mimicking extended-release formulations used in dogs (Savadelis et al., 2022), to evaluate the *D. immitis* NSG and NXG mouse models as a preventative drug screen. We demonstrated, using multiple batches of different ML-susceptible *D. immitis* isolates, in different evaluating laboratories, that injected moxidectin mediated significant 65%−89% reductions in larvae assessed between 13 and 27 days post-exposure. Hess et al. also measured ivermectin and moxidectin responses in infected NSG mice following oral doses ranging between 0.001 and 3mg/kg given on days 0, 15, and 30 post-infection (Hess et al., 2023). Their studies determine that the MO isolate and an ivermectin-resistant JYD-34 isolate of *D. immitis* were equally sensitive to moxidectin with high but incomplete efficacy demonstrable after 0.01 mg/kg dosing. They also show high levels of ivermectin efficacy against the MO, but not JYD-34 isolate, in dose titrations ranging between 0.01 and 3 mg/kg. Thus, we conclude *D. immitis* NSG and NXG models are robustly validated by multiple independent laboratories as screening tools for assessing direct-acting nematodicidal agents over at least a 28-day infection window. Our *ex vivo* and *in vivo* drug response evaluations of *D. immitis* L3/L4 in NSG or NXG mice demonstrate the flexibility to establish this model in independent laboratories with different commercially available NSG or NXG lines. Furthermore, we determine feasibility of international shipping of live mf in dog blood to produce L3s for onward experimental infections in lymphopenic mice. Mouse infections utilizing shipped L3s may allow for increased accessibility to expand experimental *D. immitis* research out of the few specialist reference centers which maintain the full life cycle of the parasite and/or the mosquito vector.

Main limitations of our study are (1) lack of data on full permissiveness to adult infections within murine cardiopulmonary vasculature, (2) lack of validation within female lymphopenic mice, and (3) <100% achievable moxidectin preventative efficacy response. In Hess et al., evaluation periods were extended and, whilst larvae continued to grow and mature within NSG mice, a divergence in growth compared with comparative dog studies was apparent after the 1st month of infection. Furthermore, there was no evidence of immature adults arriving in the heart and lungs by 15 weeks (Hess et al., 2023). The authors conclude either physiological or anatomical deficiencies may prevent the full development of *D. immitis* in mice. However, full development of the highly-related subcutaneous filaria, *Loa loa*, is possible in both NSG and RAG2^−/−^γc^−/−^ mice after 5–6 months (Pionnier et al., 2019), and thus, it remains to be tested whether full *D. immitis* development may be achieved over an extended time frame. We selected the use of male mice due to observations that even in immunodeficient systems (Rajan et al., 1994), as well as in outbred gerbils (Ash, 1971), male-biased sex-specific susceptibility is a feature of rodent filarial infections. For future pharmacological investigations, to fully enable the characterization of interactions between sex and the drug pharmacokinetic–pharmacodynamic (PK-PD) relationship, it would be useful to assess whether *D. immitis* are able to develop within female lymphopenic mouse strains. In natural hosts, injectable formulations of moxidectin are proven to mediate 100% preventative efficacies (Savadelis et al., 2022). The substantial yet incomplete moxidectin responses in our NSG and NXG *D. immitis* models may reflect that complete efficacy evolves over an extended time period, particularly considering that ML can depot in fatty subcutaneous tissues to deliver a long-tail of systemic exposure, detectable over 1 month (Al-Azzam et al., 2007; Arisov et al., 2019). Alternatively, an immunopharmacological mode of action involving decreased immunosuppressive secretions and an activated host-immune response has been proffered as one rationale why filarial larvae are differentially sensitive to ML drugs at physiological levels *in vitro* vs. *in vivo* (Moreno et al., 2010). Thus, we currently cannot rule out a potential synergy with adaptive immune-mediated responses (such as the development of opsonising or neutralizing antibodies) contributing to the complete efficacy of moxidectin. As previously discussed, passive transfer of antibodies into lymphopenic mice may determine whether such a mechanism contributes to ML preventative efficacy at physiologically relevant dose levels.

It is widely accepted that the use of specially protected, highly sentient species in preclinical research, including cats and dogs, should be strictly minimized wherever possible. This has not been plausible for veterinary heartworm preventative R&D due to a lack of a tractable small animal laboratory model. Typical drug screening has relied on *in vitro* potency testing against *D. immitis* larvae, potentially combined with initial preclinical evaluation in a surrogate rodent filarial infection model, before deciding to proceed into experimental dog infection challenge studies. Vulnerabilities of this approach include differential drug sensitivities between larvae being tested *in vitro* vs. *in vivo*, differences in filarial species larval migration routes/parasitic niches, and variability in drug target expression/essentiality across different filarial parasite species and life cycle stages, all of which may drive artifactual efficacy information. Our models, with the international commercial supply of lymphopenic strains and shipping of mf or L3 from donating laboratories, provide universal access to accurate and facile PK-PD assessments of preventative *D. immitis* drug candidate responses against the prophylactic L3–L4 larval target. Evaluations of drug larvicidal activities over the 1st month of infection, whilst larvae are developing in subcutaneous and muscle tissue, allow for rapid assessments whilst avoiding the risk of welfare issues associated with the arrival of adult parasites in the cardiovascular system. We observed no overt welfare issues in mice after parasitism, with mice gaining weight and displaying typical behavior. If adopted, our models should accelerate drug research timelines and enable more precise dose-fractionation studies for clinical selection into cat or dog studies. We conclude that *D. immitis* immunodeficient mouse models are preliminarily established for more efficient heartworm drug discovery which may, in the future, reduce the requirements for long-term cat and dog experimentation with the risk to cause severe harm, in line with an ethos of “replacement, refinement, and reduction” of animals in scientific research.

## Data availability statement

The original contributions presented in the study are included in the article/Supplementary material, further inquiries can be directed to the corresponding author.

## Ethics statement

The study protocols were approved in the UK by LSTM & University of Liverpool Animal Welfare and Ethics Review Boards and licensed by the UK Home Office Animals in Science Regulation Unit. In the USA, studies were approved by TRS Institutional Animal Care and Use Committee.

## Author contributions

JT: conceptualization. AMar and JD: data curation. AMar, JD, SH, and JT: formal analysis. MT and JT: funding acquisition. AMar, JD, SH, AS, CF, UD, AMan, CW, and EC: investigation. AMar, JD, and SH: methodology. JT, AMo, SM, and JM: project administration. FG, SW, WH, PO'N, MT, and JT: resources. AMar, JD, and JT: writing—original draft. AMar, JD, CF, JM, SM, AMo, and JT: writing—reviewing and editing. All authors contributed to the article and approved the submitted version.

## Footnotes

^1^AHS (2016).

^2^JANVIER (2019).
