# Supplementary material for: Dirofilariasis mouse models for heartworm preclinical research
Source: Front Microbiol. 2023 Jun 22;14:1208301. doi: 10.3389/fmicb.2023.1208301 (PMC10324412; doi:10.3389/fmicb.2023.1208301)
Supplement: Supplementary file 1 [file Data_Sheet_1.docx]

Supplementary Table 1: infection success rate & yields from individual experiments in various immunodeficient mouse models inoculated with 200 *D. immitis* L3

| model | Experiment / batch L3 | Isolate / laboratory | n mice | time post inoculation (days) | n infected | % infection success | Median  yield L4  (range) |
| --- | --- | --- | --- | --- | --- | --- | --- |
| RAG2^-/-^γc^-/-^ | A | MO/LSTM | 2 | 14 | 1 | 50 | 4 (0-8) |
| RAG2^-/-^γc^-/-^ | B | MO/LSTM | 1 | 14 | 1 | 100 | 5 |
| RAG2^-/-^γc^-/-^ | C | MO/LSTM | 2 | 14 | 2 | 100 | 2.5 (2-3) |
| RAG2^-/-^γc^-/^+MPA | A | MO/LSTM | 2 | 14 | 2 | 100 | 8 (7-9) |
| RAG2^-/-^γc^-/^+MPA | B | MO/LSTM | 1 | 14 | 1 | 100 | 28 |
| RAG2^-/-^γc^-/^+MPA | C | MO/LSTM | 2 | 14 | 2 | 100 | 12 (12-12) |
| NSG | 1 | MO/LSTM | 1 | 14 | 1 | 100 | 36 |
| NSG | 2 | MO/LSTM | 4 | 14 | 4 | 100 | 7.5 (6-17) |
| NSG | 3 | MO/LSTM | 1 | 14 | 1 | 100 | 18 |
| NSG | 4 | MO/LSTM | 5 | 14 | 5 | 100 | 32 (17-47) |
| NSG | 5 | MO/LSTM | 8 | 14 | 8 | 100 | 8.5 (3-14) |
| NSG | 6 | MO/LSTM | 2 | 14 | 2 | 100 | 11 (5-18) |
| NSG+MPA | 2 | MO/LSTM | 3 | 14 | 3 | 100 | 3 (1-5) |
| NSG+MPA | 3 | MO/LSTM | 1 | 14 | 1 | 0 | 0 |
| NSG | 1 | GAIII/TRS | 5 | 14 | 5 | 100 | 68 (27-68) |
| NSG | 2 | GAIII/TRS | 5 | 21 | 5 | 100 | 33 (24-58) |
| NSG | 3 | GAIII/TRS | 5 | 28 | 5 | 100 | 58 (38-59) |
| NSG | 4 | GAIII/TRS | 5 | 14 | 6 | 100 | 52.5 (30-161) |
| NXG | 1 | MO/LSTM | 2 | 14 | 2 | 100 | 2.5 (2-3) |
| NXG | 2 | MO/LSTM | 2 | 14 | 2 | 100 | 10 (8-12) |
| NXG | 3 | MO/LSTM | 5 | 14 | 5 | 100 | 22 (14-46) |
| NXG | 4 | MO/LSTM | 2 | 14 | 2 | 100 | 7 (5-9) |
| NXG | 5 | MO/LSTM | 3 | 14 | 3 | 100 | 12 (7-13) |
| NXG | 6 | MO/LSTM | 2 | 14 | 2 | 100 | 12 (8-16) |
| NXG | 7 | MO/LSTM | 2 | 14 | 2 | 100 | 9 (4-13) |

Supplementary figure 1: motility of *D. immitis in vitro* larvae over time in culture


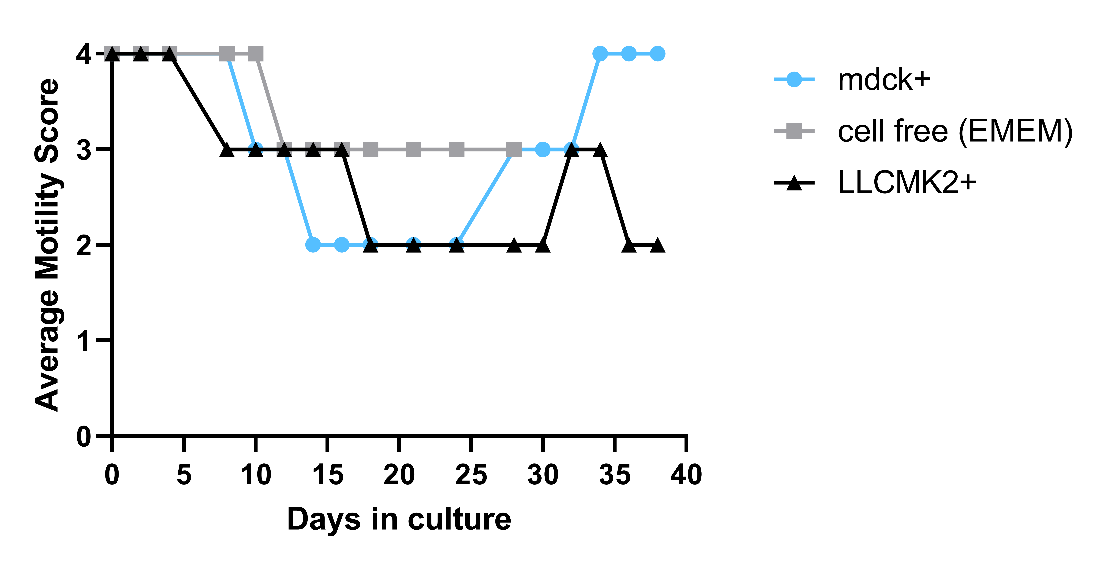


Average motility score of surviving *D. immitis* larvae (starting n=10-20) when cultured for 40 days from the infectious stage L3 with either with full EMEM media (10% FBS, 1% P/S, 1% NEAAS and 1% Amp B) or additional co-culture with Madin-Darby Canine Kidney (mdck+), or Rhesus Monkey Kidney Epithelial cells (LLCMK2+).

Supplementary Figure 2. Paralytic activity of levamisole against *in vitro* and *ex vivo D. immitis* L4 larvae

Levamisole concentration *D. immitis* motility inhibition analysis after six days exposure when using cultured *D. immitis* for periods between 15-21d L4 (red) or 15-21d L4 derived from 14dpi NSG mice (black). Non-linear curves are 3-parameter least squares fit with [IC_50_] calculated in Prism 9.1.2.

Supplementary figure 3: Weight change of animals infected with *D. immitis* L3

NSG mouse weight in grams from the point of infection (Day 0) until necropsy and parasite retrieval at either 2-, 3- or 4-weeks post infection. All mice were subcutaneously infected with 150-200 GA3 *Di*L3 at Day 0 and dosed with indicated regimens commencing the morning after infection.
